# Renal Transplantation in Systemic Lupus Erythematosus: Outcome and Prognostic Factors in 50 Cases from a Single Centre

**DOI:** 10.1155/2014/746192

**Published:** 2014-06-11

**Authors:** Ernesto Cairoli, Carolina Sanchez-Marcos, Gerard Espinosa, Constanza Glucksmann, Guadalupe Ercilla, Federico Oppenheimer, Ricard Cervera

**Affiliations:** ^1^Department of Autoimmune Diseases, Hospital Clínic, Villarroel 170, 08036 Barcelona, Catalonia, Spain; ^2^Unidad de Enfermedades Autoinmunes Sistémicas, Clínica Médica “C”, Hospital de Clínicas, Universidad de la República, Montevideo, Uruguay; ^3^Department of Nephrology and Renal Transplantation, Hospital Clínic, Barcelona, Catalonia, Spain; ^4^Department of Immunology, Hospital Clínic, Barcelona, Catalonia, Spain

## Abstract

*Background.* End-stage renal disease (ESRD) is an important cause of morbidity and mortality in patients with systemic lupus erythematosus (SLE). *Objectives.* To analyze the outcome and prognostic factors of renal transplantation in patients with ESRD due to SLE from January 1986 to December 2013 in a single center. *Results.* Fifty renal transplantations were performed in 40 SLE patients (32 female (80%), mean age at transplantation 36 ± 10.4 years). The most frequent lupus nephropathy was type IV (72.2%). Graft failure occurred in a total of 15 (30%) transplantations and the causes of graft failure were chronic allograft nephropathy (*n* = 12), acute rejection (*n* = 2), and chronic humoral rejection (1). The death-censored graft survival rates were 93.9% at 1 year, 81.5% at 5 years, and 67.6% at the end of study. The presence of deceased donor allograft (*P* = 0.007) and positive anti-HCV antibodies (*P* = 0.001) negatively influence the survival of the renal transplant. The patient survival rate was 91.4% at the end of the study. Recurrence of lupus nephritis in renal allograft was observed in one patient. *Conclusion.* Renal transplantation is a good alternative for renal replacement therapy in patients with SLE. In our cohort, the presence of anti-HCV antibodies and the type of donor source were related to the development of graft failure.

## 1. Introduction

Systemic lupus erythematosus (SLE) is the prototype of systemic autoimmune disease characterized by widespread immunologic abnormalities and multiorgan involvement including the skin, joints, lungs, heart, central and peripheral nervous system, and kidney [1]. In fact, SLE may be considered as a syndrome rather than a single disease [2].

Considering renal involvement, 40% of the SLE patients have lupus nephritis at some stage of their disease [3]. However, the prevalence of lupus nephritis varies around the world with higher rates observed in some ethnic groups, including Mestizos [4], African American, Hispanics living in the United States, and Asian compared with Caucasian [5].

Lupus nephritis is an important cause of morbidity and mortality in patients with SLE [6–8]. Of the different pathological classes, diffuse proliferative glomerulonephritis (class IV) has the worst prognosis, and end-stage renal disease (ESRD) develops in a range from 3.5 to 17% [5, 9–11]. Ethnicity, male sex, younger age, high activity histopathologic degree, interstitial fibrosis, impaired renal function at presentation, arterial hypertension as well as delay in treatment, and poor compliance are some of the unfavorable prognostic factors for ESRD in patients with lupus nephritis [12].

Recent surveys indicate that renal transplantation is associated with good outcomes in patients with ESRD due to lupus nephritis that are, in general, similar to transplant recipients with ESRD due to other causes [13, 14]. Of note, some factors of the recipient have been associated with poor outcome such as the black race, the positivity of anti-phospholipid antibodies (aPL), the peritoneal dialysis, the poor clinical conditions at the time of transplantation, and the poor treatment compliance [13, 14]. In addition, longer pretransplantation dialysis period was associated with more acute rejection in a series of Chinese SLE patients [15]. Recurrent lupus nephritis after kidney transplantation occurs in a range from 0% to 30% according to the clinical or histopathologic definition [16–18] but graft loss occurs because recurrent lupus nephritis is rare [13, 14, 19].

The objective of this study was to analyze the outcome and prognostic factors of renal transplantation in patients with ESRD due to SLE from our center.

## 2. Methods

### 2.1. Patients

We examined the medical records of patients diagnosed as having SLE whose cause of ESRD (defined as the need of chronic dialysis therapy or kidney transplantation) was primarily lupus nephritis, who required renal transplantation from January 1986 to December 2013. All patients have been systematically assessed at the Department of Autoimmune Diseases and the Department of Nephrology and Renal Transplantation of Hospital Clinic. All patients fulfilled four or more of the 1982 revised classification criteria for SLE of the American College of Rheumatology [20]. In all cases, histological class of lupus nephritis was defined according to the International Society of Nephrology/Renal Pathology Society (ISN/RPS) 2003 classification system [21]. 

### 2.2. Variables

From the patients' records, we have documented the following data: gender, age at onset of SLE, onset of clinical renal disease, and time between SLE diagnosis and lupus nephritis and between lupus nephritis and onset of dialysis. Antinuclear antibodies and aPL status, including anti-cardiolipin antibodies (aCL) and lupus anticoagulant (LA), anti-hepatitis B (HBV) and C virus (HCV), and anti-human immunodeficiency virus (HIV) antibodies, were also collected. Finally, SLE treatment prior to ESRD, duration and modalities of dialysis prior to transplantation, date of transplantation, age at transplantation and time between lupus nephritis and transplantation, donor source, posttransplantation immunosuppressive therapy used (especially the use of prednisone, mycophenolic acid, cyclosporine A, and tacrolimus), follow-up time after transplantation, lupus relapse rate and graft, and patient survival were recorded. Regarding immunosuppressive treatment, it was the same for SLE and no SLE patients. Cyclosporine A, tacrolimus, and mycophenolic acid were used according to the transplant era. Induction therapy with anti-lymphocytesantibodies was used according to the anti-HLA immunological risk.

We determined flare-ups of lupus activity and recurrence of lupus nephritis by clinical and laboratory variables. Graft failure was defined as the need to restart chronic dialysis therapy or retransplantation. 

### 2.3. Statistical Analysis

Qualitative variables are shown by frequency distributions. Quantitative variables are summarized as a mean ± standard deviation (SD). Kolmogorov Smirnov test was used for evaluation of normality. A comparison of demographic and clinical characteristics between groups (i.e., graft failure and functioning graft) was performed using Mann-Whitney *U*-test and for categorical data Fisher's exact test was used. Patient and graft survival rates were calculated with Kaplan-Meier survival curves. Patient deaths with a functioning graft were censored for the graft survival analysis. All statistical tests were two sided and assessed at *P* = 0.05 significance level. Statistical analyses were performed using SPSS software, version 20.0.

## 3. Results

In the above mentioned period, a total of 3274 renal transplantations were performed in our hospital, 50 (1.5%) of them in 40 SLE patients (32 female (80%)). Overall, 29 transplantations were from a deceased donor whereas 21 were from living donor. In 34 (68%) cases, a first transplantation was performed and in twelve (24%) and four (8%) cases, a second and a third transplantation were performed, respectively. The main demographic and clinical characteristics, histological class of lupus nephritis, immunologic features, and treatments are described in Table 1.

### 3.1. Renal Graft Survival Rates

**Figure 1:**
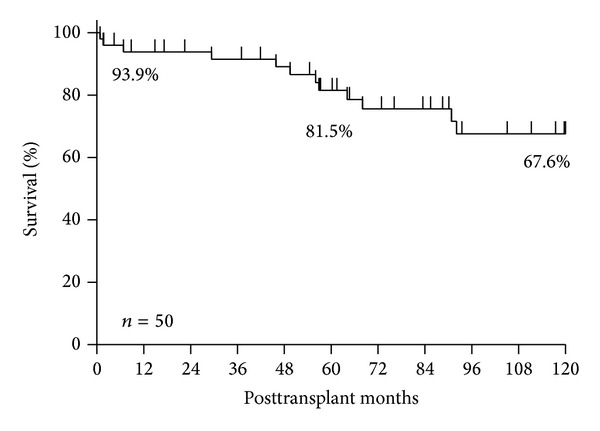
Death-censored graft survival rates at 1, 5, and 10 years.

The death-censored graft survival rates were 93.9% at 1 year, 81.5% at 5 years, and 67.6% at the end of the study (Figure 1). Clinical recurrence of lupus nephritis in renal allograft was observed in only one patient in form of membranous glomerulonephritis and chronic allograft nephropathy. Graft failure occurred in a total of 15 (30%) transplantations and the causes of graft failure were chronic allograft nephropathy (*n* = 12), acute rejection (*n* = 2), and chronic humoral rejection (*n* = 1).

### 3.2. Patient Survival Rates

The patient survival rates were 97.9% at 1 and 5 years and 91.4% at the end of the study. Four patients died at 17.6, 11, 10, and 9.4 years of the first renal transplantation, respectively. The first case was a woman who received three renal transplantations, dying as a result of* Pseudomona aeruginosa* sepsis. The second deceased patient was a woman with cirrhosis and HCV chronic infection who received two renal transplantations, dying as a result of* E. coli* sepsis. The third patient developed a coronary artery disease and died as a complication of this pathology. Finally, the forth one was a man who died because of a dilated myocardiopathy.

### 3.3. Comparison between Patients with Graft Failure versus Those with Functioning Grafts

When patients with graft failure versus functioning graft at time of the study were compared, we did not find significant differences in gender, age at SLE diagnosis, dialysis modality, and age at transplantation (Table 2). Of note, time on dialysis was longer in patients with graft failure (73.9 ± 60.6 versus 35.7 ± 35.4, *P* = 0.011). Conversely, the mean elapsed time between diagnosis of lupus nephritis and start of dialysis was higher in those patients with functioning grafts (88.7 ± 80.6 versus 39.0 ± 45.5,  *P* = 0.038). Graft failure was significantly higher in patients receiving a kidney from a deceased donor compared to living donors (*P* = 0.007, OR 10.0, 95% confidence interval [CI] 1.62–62.85) (Table 2).

As posttransplant immunosuppression therapy, all patients received prednisone and different immunosuppressive therapies (Table 2). The election of the different immunosuppressive treatment was related to the working protocol used in this moment in nephrology and renal transplant unit. Although the differences in the outcome could be related to a multifactorial origin, the majority of patients with graft failure were in the cyclosporine era. In fact, the majority of renal transplantations with graft failure were transplanted before 1998 (53% versus 17%; *P* = 0.036).

No patient had antibodies against HIV. Positive anti-HCV antibodies were detected in 22 (44%) patients; one of them was simultaneously positive for hepatitis B virus (chronic infection). The number of patients with HCV positive serology was significantly higher in the group of patients who had graft failure, whereas in 12 of them, the transplant outcome was toward the graft failure. Studies of association between graft loss and the presence of HCV positive serology showed a positive association (*P* = 0.001, OR 12.5 CI 95% [2.50–63.34]) (Table 2). When association studies were performed considering the type of donor source (deceased or living donor) and HCV positive serology, both remained as statistical significant prognostic factor of graft failure.

### 3.4. Retransplantation Cases

The retransplantation cases were analyzed separately from the main group. Overall 16 additional transplantations were performed (7 from a deceased donor and 9 from a living donor). In all cases, the initial lupus nephropathy was type IV. There were 6 graft failures whose causes were chronic allograft nephropathy (*n* = 5) and acute rejection (*n* = 1). In one patient with negative aPL and chronic allograft nephropathy, renal arterial and venous thrombosis involving medium-sized vessel wall were observed.

### 3.5. Anti-phospholipid Antibodies and Renal Transplantation

Nineteen patients (48%) had at least two aPL determinations, 12 (63%) of them being positive (5 with IgG aCL plus LA, 4 with IgG aCL only, 2 with IgM aCL plus LA, and one with LA plus IgM plus IgGaCL), and only two of them had antiphospholipid syndrome. Within this group, one of the patients that previously received two renal transplantations suffered graft loss due to intraparenchymal graft thrombosis. In another case, a patient suffered the loss of two consecutive grafts due to thrombotic microangiopathy. In both patients, previous studies were negative for aPL, starting to be positive just before the third renal transplant.

## 4. Discussion

In the present study, we have found a graft survival rate of 93.9% at 1 year, 81.5% at 5 years, and 67.6% at the end of the study and the patient survival rates were 97.9% at 1 and 5 years and 91.4% at the end of the study. These observations are similar to those reported in other recent studies from other single centers including patients from different ethnicities [22–27]. The main cause of graft failure was chronic allograft nephropathy, which is similar to data previously reported for SLE patients and also for non-SLE transplant recipients [28].

Currently, graft and patient survival of SLE patients undergoing renal transplantation are similar to those found in renal transplant recipients from other causes. These concepts are supported by the results of the European Transplant Registry and by a cohort of patients in the United States (United States Renal Data System) [13, 29]. However, other authors describe different results with lower graft survival and increased mortality in patients with SLE [30]. This difference may be explained at least partly, by methodological differences between studies in terms of prospective or retrospective design, inclusion criteria, control group, and different time of renal transplantation or recruitment period. Moreover, a retrospective study analyzed 8001 patients with SLE and renal transplantation showed that graft and patients survival were higher in those patients who received a preemptive renal transplantation compared with those who were treated with hemodialysis previously (hazard ratio [HR] 0.69; 95% CI 0.55–0.86, *P* < 0.01 versus HR 0.52; 95% CI 0.38–0.70, *P* < 0.01, resp.) [31]. In fact, in the current series, time on dialysis was significantly shorter in patients with functioning graft. Thus, as in other diseases with ESRD, renal transplantation is considered the procedure of choice for renal replacement therapy in patients with SLE [31].

In our series, relapsing lupus nephritis was found only in one case (2%). The recurrence rate of lupus nephritis was reported initially to be around 1–4% [32, 33]. However, immunofluorescence and electron microscopy studies performed in renal biopsies of SLE transplanted patients detected a rate of recurrent lupus nephritis of 30% [19, 34, 35]. However, it does not seem to negatively affect allograft or patient survival [19, 34]. Interestingly, Norby et al. [17] found a recurrence of lupus nephritis in 54% of renal biopsies from 41 SLE patients with renal transplant. However, the majority of them were subclinical in form of histological class I or II. Of note, 83% of the transplanted kidneys presented with signs of chronic allograft nephropathy, regardless of the presence or absence of lupus nephritis. Similar results of recurrence of lupus nephritis have been described in a Chinese kidney transplant cohort of 32 SLE patients [22].

Our results showed that factors that negatively influenced the survival of the renal transplant were the presence of deceased donor allograft (*P* = 0.007), positive anti-HCV antibodies (*P* = 0.001), and a longer time on dialysis before transplantation (*P* = 0.011). In retrospective studies performed on databases, the deceased donor allograft recipients have worse outcomes compared with living allograft recipients [30] and African American and Caucasian Americans have similar allograft failure rates [36].

A particular feature of this series is the high number of patients with HCV infection, mainly located in the group of transplant failure, showing a significant positive association with the lower graft survival (OR 12.5, 95% CI 2.50–63.34), in the same manner as that described in non-SLE patients [37, 38]. Recent evidence documents that the concomitant HCV infection in patients with lupus nephritis is associated with worse renal outcome, higher rate of progression to ESRD, and reduced patient survival [39]. In a retrospective study involving 1624 patients with positive serology for HCV undergoing kidney transplant, Batty et al. [40] found a higher mortality (HR 1.23; 95% CI 1.01–1.49, *P* = 0.04) and higher rate of hospitalization in patients positive for HCV compared with patients serologically negative. A recent systematic review collecting 18 series described the negative impact of HCV infection in the outcome of renal transplantation, with increased mortality (HR 1.69; 95% CI 1.33–1.97, *P* < 0.0001) and graft loss (HR 1.56; 95% CI 1.22–2.004, *P* < 0.0001) [41]. However, in the last two studies [40, 41], lupus nephropathy was not specifically analyzed. Although the intimate pathogenic mechanisms by which HCV induces a negative impact on renal graft remain to be known, there is some evidence attributing to plasmatic viremia and anti-HCV antibodies themselves a possible pathogenic role impairing the kidney function or inducing the development of chronic nephropathy allograft [37, 42].

The reason why HCV recipients are overrepresented in this cohort of patients is probably related to the high rate of repeated transplantations. Twenty-two transplants in HCV positive recipients were distributed between 13 patients: 5 patients with one, 7 patients with two, and one patient with three transplants. By contrast within the 28 transplants in HCV negative recipients, there were 26 patients with one transplant and one patient with two transplants. Many of those HCV positive patients initiated dialysis therapy before the HCV screening test was available.

In our series, the use of mycophenolic acid, tacrolimus, and negative aPL determinations seem to be related with better renal graft survival, supporting the possible multifactorial origin of the improved performance. Moreover, thanks to methodological advances in transplantation procedure, the use of mycophenolic acid and tacrolimus in recent years partly explain the significant differences found in our series, thus, supporting the benefit of their use.

As shown in our series, coronary artery disease was one of the causes associated with mortality in the outcome. Recent studies demonstrate a reduction in cardiovascular risk with the administration of fluvastatin in patients with lupus recipients of kidney transplantation [43]. Two more patients died because of sepsis, probably related to immunosuppressive treatment.

Thrombotic events have been reported more frequently in renal transplantation recipients with aPL worsening their functional prognosis [14, 23]. In a recent study, the presence of LA at the time of renal transplantation was associated with a high rate of allograft nephropathy associated with antiphospholipid syndrome and poor transplantation outcomes [44]. In the current series, aPL determinations were available in 19 patients, because the systematic screening in the renal transplant unit was carried out only in recent years. In the present series, the allograft failure was related to thrombosis and thrombotic microangiopathy associated with the presence of aPL in two cases; therefore their detection as well as their repetition in the time, despite their negativity, should be recommended in the pretransplantation period.

Current study had some limitations. Due to the retrospective design of our analysis, some points such as the role of activity of SLE in the graft failure or the role of sociodemographic and environmental factors such as educational level, socioeconomic status, or smoking could not be analyzed. Moreover, the limited number of SLE patients who received kidney transplantation is the reason why some significant associations should be considered with caution as indicated by the wide range of confidence intervals. In the data collected, the number of patients with aPL determinations performed before or at the time of kidney transplantation was low; therefore the association between these antibodies and the thrombotic complications was weak and not significant.

Renal transplantation is a good alternative for renal replacement therapy in patients with SLE, but the existence of HCV positive serology and a thrombotic disease associated with the aPL could be related to the development of graft failure. In our series, the patient and graft survival rates as well as factors associated with these end points are similar to that of ESRD caused by other diseases.

## Figures and Tables

**Table 1:** Demographic and clinical characteristics, histological and immunologic features, and treatments used in the cohort of SLE transplanted patients.

Demographic characteristics	
Gender female	32 (80%)
Ethnicity	
Caucasians	38 (95%)
Hispanics	2 (5%)
Age at SLE diagnosis (years)	22.7 ± 10.5
Age at renal transplantation (years)	36 ± 10.4
Time between SLE diagnosis and lupus nephritis (months)	28.4 ± 65.1
Time between lupus nephritis and onset of dialysis (months)	68.8 ± 72.3
Time on dialysis (months)	50 ± 49.4
Time between diagnosis of lupus nephritis and transplantation (months)	118 ± 69
Time of followup (months)	71.4 ± 41
Histological diagnosis at onset of lupus nephritis:	
Type IV	26 (72%)
Type III	3 (8%)
Type II	2 (5%)
Type V	2 (5%)
Type VI	1 (3%)
Interstitial nephritis	1 (3%)
Thrombotic microangiopathy	1 (3%)
Unknown	4 (10%)
Number of transplantations	
First transplantation	34 (68%)
Second transplantation	12 (24%)
Third transplantation	4 (8%)
Donor source	
Cadaveric donor	29 (58%)
Living donor	21 (42%)
HLA identical siblings	4 (19%)
Other genetically related	13 (62%)
Unrelated donors	4 (19%)
Immunologic features at renal transplantation	
Antinuclear antibodies	50 (100%)
Anti-dsDNA antibodies	30 (60%)
Anti-phospholipid antibodies	12 (63%)
Treatments	
Cyclosporine/tacrolimus	19/27
Azathioprine/mycophenolic acid	6/38
Sirolimus	3
ATG/OKT3/Basiliximab/no induction	23/1/9/17
Graft failure (%)	15 (30%)

Quantitative variables are presented as mean ± standard deviation and qualitative variables as number (percentage). Treatments are presented as number of transplantations.

SLE: systemic lupus erythematosus; ATG: antithymocyte globulin; OKT3: orthoclone.

**Table 2:** Comparison of demographic features, clinical characteristics and treatment between SLE patients with graft failure and functioning graft.

	Graft failure (*n* = 15)	Functioning graft (*n* = 35)	*P*
Gender female (%)	14 (93%)	27 (77%)	0.169
Age at diagnosis SLE (years)	22.4 ± 10	22.8 ± 11	0.758
Age at renal Tx (years)	41.3 ± 10.2	38.7 ± 12.0	0.280
Time SLE-nephritis (months)	17 ± 42.6	34.9 ± 75	0.412
Time nephritis dialysis (months)	39 ± 45.5	88.7 ± 80.6	0.038
Time on dialysis (months)	73.9 ± 60.6	35.7 ± 35.4	0.011
Time nephritis-Tx (months)	114.6 ± 64.2	120 ± 73.3	0.880
Dialysis before renal Tx (%):			
HD	14 (93.3%)	19 (76.0%)	0.168
CAPD	2 (13.3%)	7 (28.0%)	0.251
HD and CAPD	1 (6.7%)	3 (12.0%)	0.516
Tx date (years)	1998 ± 7	2004 ± 6	0.036
Donor source (%):			
Cadaveric	13 (86.7%)	16 (45.7%)	0.007
Living donor	2 (13.3%)	19 (54.3%)	—
Immunosuppressive regimen at Tx (grafts) (%):			
Cyclosporine A	10 (66.6%)	9 (25.7%)	0.006
Mycophenolic acid	8 (53%)	31 (88.6%)	0.003
Tacrolimus	4 (27%)	23 (66%)	0.012
Positive anti-HCV antibodies (patients) (%)	12 (80%)	10 (28.6%)	0.001
Positive aPL antibodies (%)	1 (6.7%)	11 (31.4%)	0.058

Quantitative variables are presented as mean ± standard deviation and qualitative variables as number (percentage).

SLE: systemic lupus erythematosus; Tx: transplantation; HD: hemodialysis; CAPD: continuous ambulatory peritoneal dialysis; HCV: hepatitis C virus; aPL: anti-phospholipid antibodies.
